# The serological diagnosis of coeliac disease - a step forward 

**Published:** 2018

**Authors:** Geoffrey Holmes, Carolina Ciacci

**Affiliations:** 1 *Royal Derby Hospital, Derby, UK *; 2 *Coeliac Center at Department of Medicine and Surgery, Scuola Medica Salernitana, University of Salerno, Salerno, Italy *

**Keywords:** Celiac disease, Tissue transglutaminase, Serology, Diagnosis

## Abstract

The development of highly performing serological tests to identify patients with coeliac disease (CD), allowed large scale screening studies to be carried out and the results transformed our understanding of the prevalence of the condition in the general population. The next logical step was to ask whether CD could be reliably diagnosed by these tests without the need for small intestinal biopsies. This was shown to be the case. Studies from Derby, UK, indicated that about half of adult patients can be diagnosed in this way and similar figures have been provided for children. When considering this approach, it is essential that laboratories only use highly performing test kits that they have validated to measure tissue transglutaminase antibodies because all kits do not function to the same high standard. There remains a place for biopsy when criteria for serological diagnosis are not met, if the diagnosis of CD is strongly suspected but serological tests are negative or in patients not showing the expected responses to gluten free diet or otherwise causing concern, when not only small bowel biopsy will be indicated but also other investigations. Those with refractory CD should not be compromised by this diagnostic strategy. As serological tests become more refined and information accumulates, it is likely that this mode of diagnosis will gather momentum for the benefit of patients and carers. This brief review looks at the evidence for making the diagnosis of CD in some cases by serological tests alone.

## Introduction

 In the 1950s, the demonstration that contrary to previous belief, the mucosa of the small intestine was abnormal in patients with coeliac disease (CD) and the discovery that gluten, a protein complex in wheat, rye and barley was the damaging agent, revolutionised diagnosis and management and allowed more precise definition. Definitions have revolved around findings in the small bowel mucosa, responses to gluten withdrawal and challenge and associated clinical reactions. From the first however, matters were not as clear cut as initially might appear. For example, what constitutes an abnormal mucosa? What can be taken as a positive morphological response to a gluten free diet? How can a clinical response be judged in patients who have mild or even no symptoms? All of these uncertainties were reflected in definitions put forward through the years. Some workers required a flat biopsy (1) but others only an abnormal mucosa (2). Some definitions required demonstrating a deterioration in mucosal architecture as a result of gluten challenge (3). Others wanted a dramatic clinical improvement on gluten free diet (1) while yet others took no account of symptoms. Forty-five years ago with regard to dermatitis herpetiformis, an excess of intraepithelial lymphocytes in otherwise normal villi was taken as evidence of CD (4). How to define CD has generated much heated debate but it is now regarded as a chronic small intestinal immune-mediated enteropathy precipitated by exposure to dietary gluten in genetically susceptible people (5). 

In the early years, from the practical point of view the diagnosis of CD in those presenting with classical symptoms of weight loss, diarrhoea and fatty stools indicative of malabsorption, a flat mucosa and who improved on gluten free diet was straightforward and secure. How many, who presented with so-called atypical or mild symptoms and had only minor mucosal changes, were misdiagnosed will never be known. Over-diagnosis and under-diagnosis must have occurred. This uncertainty was one of the factors that spurred researchers to look for other indicators of CD and they turned their attention to possible blood markers. 

**Antigliadin antibodies**

Logic dictated that antigliadin antibodies would be found in the serum and reduce following dietary gluten withdrawal. This proved to be the case (6). In a study of 61 patients with untreated CD and a large group of controls the sensitivity for the test was 93% and the specificity 95% (6). The predictive value of a positive test was found to be 50%. After 2 years on a gluten free diet normal antigliadin antibody concentrations were observed. The test could therefore assist clinicians in deciding who to biopsy and provided objective evidence of compliance with gluten free diet but was less than ideal. More recently attention has turned to the use of antibodies against deamidated gliadin peptides which may have a role in a non-biopsy diagnostic strategy for CD (7).

**Figure 1:**
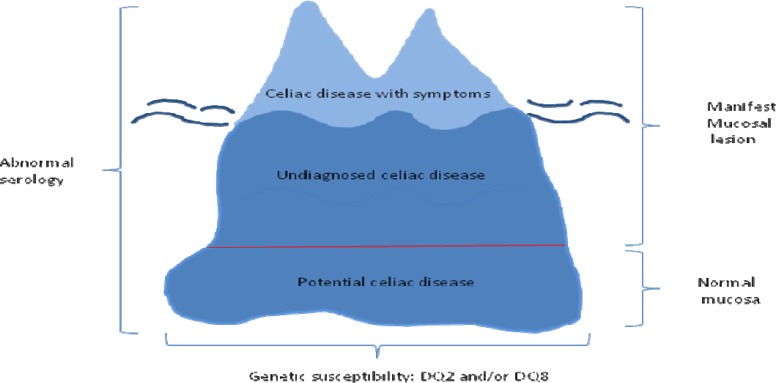
The celiac iceberg. For each patient with celiac disease diagnosed on clinical grounds there are many others that remain undiagnosed, shown by the submerged of the iceberg, because of an atypical presentation, lack of symptoms or the potential stage of the disease.

**Endomysial and tissue transglutaminase antibodies for screening**

In 1983, endomysial antibodies were found in the serum of patients with CD and dermatitis herpetiformis (8). With very high sensitivity and specificity it was almost an ideal diagnostic test (9). In 1997, the enzyme tissue transglutaminase was identified as the endomysial antigen which led to the development of a further test that importantly could be quantified (10) and had high diagnostic accuracy (11). These advances made it possible to perform large scale population screening studies which indicated that CD was much more common than hitherto supposed. Such an investigation in England revealed a serological prevalence of approximately 1% (12), a figure found in other populations (13, 14) but most patients are undiagnosed. This information gave rise to the concept of the coeliac iceberg with the majority of patients undiagnosed, represented by the submerged portion (figure 1) (15). 

**Serological tests for diagnosis**

When it was shown that serological tests could be used satisfactorily in screening studies to detect CD, it was logical to ask whether it might be possible to establish the diagnosis using these tests alone (16-19). A study in Derby showed that it was feasible in adult patients to define a level of tissue transglutaminase antibodies above which the positive predictive value for CD was 100% (20). A further study carried out in Derby verified this view (21). Taken together, these two studies included 493 patients with CD, 259 (52.5%) of whom had tissue transglutaminase antibodies above the cut-off and would have avoided biopsies (22). Numerous investigations have supported the view that a biopsy can be avoided in many adult patients (23-32) and children (7, 26-29, 31, 33-37) by employing serological tests. An investigation from North America involving 4 centres concluded that tissue transglutaminase levels could not be relied on and biopsies were necessary to make the diagnosis of CD. However, this was a retrospective study and 3 of the centres contributed only small numbers of cases raising the issue of patient selection (38). There was also lack of test standardisation. A recent, important paediatric survey employed prospective data on 707 subjects 18 years of age or younger, gathered from 33 gastroenterology units in 21 countries (36). It was found that 399 (56.4%) qualified for the non-biopsy approach according to ESPGHAN guidelines (39). 

The case for the serological diagnosis of CD is so strong that the principle has been incorporated into ESPGHAN guidelines (39) which have been validated in retrospective and prospective studies (40-44) and may also be applicable to children without symptoms (45). A Canadian retrospective appraisal of the guideline criteria found 98.2% of children could be diagnosed without an intestinal biopsy that would have resulted in a 50% reduction in endoscopies performed to evaluate CD per year (37). In this study, four children who met the criteria were found not to have CD although one subsequently developed villous atrophy and another villous blunting. Repeat biopsies were not carried out on the other two. When relatively high titres of endomysial antibodies (≥ 1:80) were evaluated in both symptomatic and asymptomatic children, all had biopsies consistent with CD (37). Some patients who are EMA positive but have normal biopsies are termed potential coeliacs (46). These would be diagnosed because they would have antitissue transglutaminase levels below the cut-off to accept the diagnosis of CD on serological grounds and so require a biopsy. In some instances, this would be normal so identifying those with potential CD.

The British Society of Gastroenterology Guidelines for the diagnosis of CD maintain that a biopsy is essential for diagnosis and cannot be replaced by serology (47). This advice ignores much evidence to the contrary and has been criticised (48, 49). Although regarded by some as the gold standard, small bowel biopsies are not without problems. About 10% of specimens cannot be reliably interpreted because of poor preparation (50). In order not to miss the diagnosis of CD it is necessary to take biopsies also from the duodenal bulb (51). Taking biopsies from this area has not caused major problems in most studies (52) but in one report, 45% were unsatisfactory for diagnosis because they were small, superficial or fragmented and a hard mucosal lining was cited as the cause (53). Such difficulties can be largely overcome by attention to detail such as making sure forceps retrieve a generous pieces of mucosa rather than just scratch the bowel surface. It also has to be recognised that histopathologists vary in their interpretation of small bowel biopsies and lesions can be patchy, leading to the misdiagnosis of CD (36, 54-57). 

When considering serological diagnosis there are some important considerations to take into account. People should not be put on a gluten free diet just because tissue transglutaminase antibodies are positive. Consideration of positive serology alone is not enough. Titres or levels in terms of the upper limit of normal for the test being used have to be taken into account and it has been shown that above these limits, small intestinal histology is consistent with the diagnosis of CD. In the measurement of tissue transglutaminase, laboratories must use a high performing test kit that they have validated, for not all tests perform to the same high standard and should not be used (28, 58). Endomysial antibodies should be measured to guard against false positive tissue transglutaminase antibody results and this is included in the Derby diagnostic algorithm (21). False positive tissue transglutaminase antibody results may occur when polyclonal IgA is increased as in chronic liver disease or in patients with an IgA monoclonal gammopathy (11). However, false positive results of a magnitude that would lead to an incorrect diagnosis of CD has never been encountered using the Derby algorithm. 

False negative tissue transglutaminase as a result of IgA deficiency can be avoided by measuring serum IgA in children 12 years and younger and in any patient with very low IgA antibodies when an IgG based test should be used (21).

Individuals with villous atrophy who have negative serological tests for CD despite normal levels of IgA, should undergo further investigation without initiating gluten free diet (59). In an Italian study of 810 adult patients with CD the prevalence of seronegative disease was 1.7% (59). A UK investigation that evaluated 200 adult patients with seronegative villous atrophy showed that CD accounted for 31% of cases, while infections, peptic duodenitis, drugs and immunological disorders were among other major causes (60). In the future, routine assessment for the presence of intestinal markers of gluten enteropathy, such as tissue transglutaminase 2 or TCR-γδ/CD3+, will better characterize seronegative patients (61). In patients with seronegative CD it is recommended that the diagnosis is supported by HLA testing and resolution of symptoms and improvement of small bowel mucosal architecture on gluten free diet. 

**HLA markers and diagnosis**

The determination of HLA status can be used to support the diagnosis of CD while those who lack the markers HLA-DQ2 and HLA-DQ8 are unlikely to develop the condition (35, 39). 

**Role for small intestinal biopsy**

It is important to understand that no one is saying that small bowel biopsy is obsolete. It still has a crucial part to play in the diagnosis of CD when the serological criteria are not met, if the diagnosis is strongly suspected but serological tests are negative or in those not showing the expected response to gluten free diet. If clinicians suspect that they are not dealing with a straightforward patient with CD, whatever the tissue transglutaminase antibody level, biopsy and other tests are indicated. Patients with refractory coeliac disease (RCD) should not be compromised by a strategy that recognises serological diagnosis. Those with RCD 1 are likely to be no more than patients who are exquisitely sensitive to gluten and when the diet is adjusted they will flourish (62). RCD 2 is rare. In the Derby coeliac clinic of 713 unselected coeliac patients reviewed some years ago, 5 (0.7%) had RCD 2 (63). In a multicentre prospective study of 1840 coeliac patients, 7 developed RCD (0.38%; 5 RCD 1 and 2 RCD 2) over an observation period of 48 months (64). These patients are markedly unwell. Clinical features and laboratory tests indicate that something is seriously amiss that requires urgent attention including endoscopy, biopsy and imaging. 

## Future considerations

In recent years there has been a movement away from morphological to serological criteria, to establish the diagnosis of CD and as serological tests are refined and more information accumulates, this is likely to accelerate. A multicentre study using a standardised approach is now required to further explore the serological cut-off that predicts CD in a large number of patients. Such an investigation will also allow the role of endomysial antibodies in a serological diagnosis strategy to be determined. Serological tests for CD are among some of the best performing tests in medicine and gastroenterologists are fortunate to have them available. These have transformed knowledge regarding the prevalence of CD and have aided the diagnosis immeasurably but still have to be used intelligently. 

## Conflict of interests

The authors declare that they have no conflict of interest.
